# Immunomodulatory Effects of Bone Marrow-Derived Mesenchymal Stem Cells on Pro-Inflammatory Cytokine-Stimulated Human Corneal Epithelial Cells

**DOI:** 10.1371/journal.pone.0101841

**Published:** 2014-07-08

**Authors:** Li Wen, Meidong Zhu, Michele C. Madigan, Jingjing You, Nicholas J. C. King, Francis A. Billson, Kathryn McClellan, Gerard Sutton, Con Petsoglou

**Affiliations:** 1 Save Sight Institute & Discipline of Clinical Ophthalmology, University of Sydney, Sydney, New South Wales, Australia; 2 Lions New South Wales Eye Bank, NSW Organ and Tissue Donation Service, Sydney, New South Wales, Australia; 3 Sydney Eye Hospital, Sydney, New South Wales, Australia; 4 School of Optometry & Vision Science, University of New South Wales, Kensington, New South Wales, Australia; 5 Discipline of Pathology, School of Medical Sciences and Bosch Institute, Sydney Medical School, University of Sydney, Camperdown, New South Wales, Australia; 6 Sight for Life Foundation, Sydney, New South Wales, Australia; 7 Vision Eye Institute, Chatswood, New South Wales, Australia; 8 Auckland University, Auckland, New Zealand; Georgia Regents University, United States of America

## Abstract

**Purpose:**

To investigate the modulatory effect of rat bone marrow mesenchymal stem cells (MSC) on human corneal epithelial cells (HCE-T) stimulated with pro-inflammatory cytokines interferon gamma (IFN-γ) and tumor necrosis factor alpha (TNF-α) in an *in vitro* co-cultured model.

**Methods:**

HCE-T alone and co-cultured with MSC were stimulated with IFN-γ/TNF for 24 and 48 hours or left untreated. The expression of intracellular adhesion molecule (ICAM)-1, human leukocyte antigen ABC, DR and G (HLA-ABC, HLA-DR, HLA-G) were investigated by flow cytometry. Subcellular localization of nuclear factor-kappa B (NF-κB) and expression of indoleamine 2,3-dioxygenase (IDO) were assessed by immunofluorescence staining and western blot. The concentration of transforming growth factor beta 1 (TGF-β1) in the conditioned media from different cultures was evaluated by enzyme-linked immunosorbent assay. NF-κB and TGF-β1 signaling pathway blocking experiments were performed to analyze associations between the expression of cell surface molecules and the NF-κB transcription pathway, and the expression of IDO and TGF-β1 signaling pathway.

**Results:**

IFN-γ/TNF treatment significantly up-regulated expression of ICAM-1, HLA-ABC, and induced *de novo* expression of HLA-DR and IDO on HCE-T cultured alone, while HLA-G expression remained unaffected. Up-regulation was significantly inhibited by co-culture with MSC. Increased TGF-β1 secretion was detected in 48 h IFN-γ/TNF-stimulated MSC monocultures and HCE-T/MSC co-cultures. MSC attenuated the activation of cytokine-induced NF-κB and IDO induction. Blockade of NF-κB transcription pathway by BMS-345541 significantly reduced the up-regulation of ICAM-1, HLA-ABC, HLA-DR and IDO expression, while blockade of TGF-β1 signaling pathways reversed the modulatory effect of MSC on IDO expression.

**Conclusions:**

MSC reduced the expression of adhesion and immunoregulatory molecules on pro-inflammatory cytokine-stimulated HCE-T via the NF-κB transcription pathway. MSC attenuated expression of IDO through both NF-κB transcription and TGF-β1 signaling pathways. Co-culture of HCEC with MSC therefore provides a useful *in vitro* model to study the anti-inflammatory properties of MSC on corneal epithelium.

## Introduction

Corneal epithelium is the outermost layer of the cornea. It maintains the integrity and transparency of the cornea and forms a barrier to protect the cornea from injury and harmful foreign agent invasion. Inflammation is part of the initial reaction of the cornea to chemical, thermal and mechanical injury and is associated with the infiltration of neutrophils, monocytes/macrophages and lymphocytes to the site of damage [1], [2], as well as generation of pro-inflammatory cytokines, such as IFN-γ/TNF, IL-1 [3]–[5]. The release of IFN-γ and TNF activates the NF-κB pathway, which in turn up-regulates a large number of target genes. These include crucial immune recognition molecules, such as major histocompatibility complex-I (MHC-I) and MHC-II, recognized by CD8^+^ and CD4^+^ T cells, respectively, and intracellular adhesion molecule (ICAM-1, CD54), recognized by the integrin, leukocyte function antigen-1 (LFA-1, CD11a/CD18), expressed on lymphocytes and many members of the myeloid lineage. Increased ICAM-1 and MHC-II expression on injured corneal epithelium further play an important role in the infiltration of activated leukocytes [6], [7]. Excessive activation of immune cells can interfere with repair, aggravating injury, eventually leading to permanent corneal damage and visual impairment [8]. However, IFN-γ and TNF also induce secretion of soluble immunomodulatory molecules, such as transforming growth factor-β1 (TGF-β1) and interleukin-10 (IL-10), which are anti-inflammatory [9], [10], as well as expression of indoleamine 2,3-dioxygenase (IDO), the first enzyme in the tryptophan metabolic pathway [11]. The IDO-mediated depletion of tryptophan and subsequent accumulation of active metabolites is strongly linked to immune suppression and in the eye, prolonged corneal allograft survival [12]. IDO may also protect corneal endothelial cells from UV-induced oxidative stress and damage [13]. Thus, these factors play dual roles in both the generation of inflammatory responses and wound healing.

Bone marrow mesenchymal stem cells (MSC) are adherent bone marrow progenitor cells with a fibroblastic morphology that distinguishes them from hematopoietic progenitor cells [14]. MSC are an attractive candidate for tissue repair and wound healing, because of their easy isolation, capacity for self-renewal, potential for multiple lineage differentiation and anti-inflammatory properties. Systemically injected in animal models of corneal injury, MSC migrate to the injury site and engraft to promote wound healing [15]. Although trans-differentiation of MSC into corneal epithelial cells is considered to play a role in tissue repair [16]–[18], studies also show that the anti-inflammatory and anti-angiogenic effects of MSC are essential in this process [19]–[21]. This is supported by evidence of the immunosuppressive and anti-angiogenic profile of *in vitro* soluble factors secreted by MSC co-cultured with damaged corneal epithelial cells [22].

To date, the mechanisms involved in the immunomodulatory effects of MSC on corneal wound healing have not been fully elucidated. In the present study, we mimicked the inflammatory environment using a combination of IFN-γ and TNF in co-cultures of MSC and corneal epithelial cells. We investigated the modulatory effects of MSC on the expression of a range of immunoglobulin superfamily (IgSF) recognition molecules normally up-regulated in inflammation, in IFN-γ/TNF-stimulated corneal epithelial cells, as well as the expression of anti-inflammatory elements such as IDO and TGF-β1, induced by these cytokines. In order to dissect the underlying mechanism(s) we further examined the effects of MSC on the activation of the NF-κB transcription and TGF-β1-signaling pathways, which are associated with this modulation. Our hypotheses, experimental design and findings are summarized in Figure S1.

## Materials and Methods

Animal ethics approval was obtained from the Animal Ethics Committee, University of Sydney (Ethic approval number: K17/12-2007/3/4757). All procedures were conducted in accordance with relevant national and international guidelines.

### Cell isolation and culture

#### 1. Mesenchymal stem cell (MSC) isolation and culture

Bone marrows from femurs and tibias of 3–4 weeks old Wistar rats were separated by density gradient centrifugation with Ficoll-Paque PLUS (Sigma-Aldrich, St. Louis, USA). The cells from the interface layer were resuspended with low glucose Dulbecco's Modified Eagles Medium (DMEM, Invitrogen, CA, USA) supplemented with 15% fetal bovine serum (FBS), 50 µg/ml of penicillin and streptomycin, and 2 mM glutamine (all reagents from Invitrogen). A final concentration of 5×10^6^ cells were seeded in 25 cm^2^ tissue culture flasks (NUNC, Thermo Fisher Scientific Inc., MA, USA), and grown at 37°C in a humidified atmosphere of 5% CO_2_. Medium was changed two days after seeding, and then twice per week until the cells reached around 80% confluence. The cells were then passaged with 0.25% trypsin/0.1% EDTA (Invitrogen) and grown in MSC Growth medium (M-GM) [DMEM-low glucose supplemented with 10% FBS, 50 µg/ml penicillin and streptomycin, and 2 mM glutamine (Invitrogen)]. Flow cytometric analysis was used to identify the molecular phenotype of rat-MSCs. Passages 3–5 were used for all experiments.

#### 2. Human corneal epithelial cell culture

A human SV40-adenovirus vector transformed corneal epithelial cell line (HCE-T, Riken Cell Bank, Japan) was used for these experiments [23]. Cells were cultured and maintained in HCE-T Growth Medium (H-GM) [1∶1 DMEM/Hams F12 supplemented with 5% FBS, 10 ng/ml human epidermal growth factor (EGF), 5 µg/ml insulin, 50 µg/ml penicillin and streptomycin and 2 mM glutamine (all reagents from Invitrogen)], according to the manufacturer's instruction.

#### 3. HCE-T and MSC co-culture

For the co-cultures system, HCE-T cells were seeded in 6 well plates; MSC cells were seeded on to permeable membrane inserts (0.45 µm pore size, 24.5 mm diameter, Transwell, Corning Incorporated, NY, USA) and grown in M-GM with FBS reduced to 5%. After 3 h, Transwells with MSC cells were placed in the 6 well plates, above the HCE-T cells.

### Treatment paradigms

The following experiments were conducted using the established cell culture models.

#### 1. Cytokine treatment

This experiment was to establish pro-inflammatory cytokine stimulation models using combined IFN-γ and TNF recombinant proteins (Table 1).

**Table 1:** Cytokine Treatment Groups.

Group 1	HCE-T only, untreated control
Group 2	HCE-T only, 24 h
	[100 U/ml rh IFN-γ +100 U/ml rh TNF]
Group 3	HCE-T/MSC, 24 h
	[HCE-T 100 U/ml rH IFN-γ +100 U/ml rH TNF]
	[MSC 100 U/ml rR IFN-γ + rR TNF 100 U/ml]
Group 4	As for Group 2, 48 h cytokines stimulation
Group 5	As for Group 3, 48 h cytokines stimulation

rH - recombinant human; rR - recombinant rat.

rH TNF-α (Sigma-Aldrich), rH IFN-γ (Sigma-Aldrich).

rR TNF-α (BioLegend, San Diego USA), rR IFN-γ (BioLegend).

After 3 days in culture, fresh media were added into monocultured HCE-T and co-cultured HCET-T/MSC cells (with EGF and insulin were omitted from H-GM). Immediately, 100 U/mL of recombinant human (rH) IFN-γ/TNF and recombinant rat (rR) IFN-γ/TNF were added to HCE-T and MSC cells respectively and incubated for 24 and 48 h (detailed in Table1).

#### 2. NF-κB pathway examination

Monocultured and co-cultured HCE-T cells were prepared as described above. After 3 days in culture, the cells were changed to fresh growth medium and treated with DMSO as vehicle and BMS-345541 a NF-κB pathway inhibitor, (5 µM, Sigma-Aldrich) for 1 h incubation, followed by IFN-γ/TNF treatments for 24 and 48 hours (Table 2, three replicates were conducted). The HCE-T cells were harvested and subject to flow cytometry and western blot analysis.

**Table 2:** Groups for NF-κB pathway.

Group 1	HCE-T only, untreated control
Group 2	HCE-T only, treated with vehicle (DMSO) 1 h, followed 24 h 100 U/ml rH IFN-γ +100 U/ml rH TNF
Group 3	HCE-T only, treated with BMS-345541 (5 µM) 1 h, followed 24 h 100 U/ml rH IFN-γ +100 U/ml rH TNF
Group 4	As for group 2, HCE-T only, treated with vehicle (DMSO) 1 h, followed 48 h cytokines stimulation
Group 5	As for group 3, HCE-T only, treated with BMS-345541 (5 µM) 1 h, followed 48 h cytokines stimulation

DMSO - Dimethyl sulfoxide (Research Organics, OH USA).

#### 3. Assessment of TGF-β1 pathway and its relationship with IDO production

HCE-T and MSC monocultures and HCE-T/MSC co-cultures were established as described above. The cells were treated as described in Table 3 (Groups 1 to 8, n = 3 repeat experiments). Enzyme-linked immunosorbent assay (ELISA) was used to examine for secreted TGF-β1 in all groups including untreated controls and cytokine-stimulated cells.

**Table 3:** Groups for detection of TGF-β1 secretion.

Group 1	HCE-T only, untreated control
Group 2	HCE-T only, 24 h 100 U/ml rH IFN-γ +100 U/ml rH TNF
Group 3	HCE-T/MSC, 24 h
	[HCE-T 100 U/ml rH IFN-γ +100 U/ml rH TNF]
	[MSC 100 U/ml rR IFN-γ +100 U/ml rR TNF]
Group 4	As for group 2, HCE-T only, 48 h cytokines stimulation
Group 5	As for group 3, HCE-T/MSC, 48 h cytokines stimulation
Group 6	MSC only, untreated control
Group 7	MSC only, 24 h 100 U/ml rR IFN-γ +100 U/ml rR TNF
Group 8	MSC only, 48 h 100 U/ml rR IFN-γ +100 U/ml rR TNF stimulation

To further study the effects of TGF-β1 in IDO regulation, we examined (a) the effects of exogenous TGF-β1 (10 ng/ml, BioLegend, CA, USA) on IDO expression in control and cytokine-treated HEC-T cells, and non-cytokine-treated HCE-T/MSC co-cultures (Table 4), and (b) the effects of blocking the TGF-β1 signaling pathway in HCE-T monocultures and HCE-T/MSC co-cultures (Table 5). SB-431542 (1 µM, Sigma-Aldrich), a TGF-β receptor I inhibitor was used together with monoclonal mouse anti-TGF-β1 antibody (1 µg/ml, R&D System, Minneapolis, USA) in the inhibition experiments (Table 5). IDO expressions were determined by western blotting.

**Table 4:** Groups for exogenous TGF-β1 treatment.

Group 1	HCE-T only, untreated control
Group 2	HCE-T only, 24 h
	[100 U/ml rh IFN-γ +100 U/ml rh TNF]
Group 3	HCE-T only, TGF-β1 10 ng/ml 1 h treatment, followed 24 h
	[100 U/ml rh IFN-γ +100 U/ml rh TNF]
Group 4	HCE-T only, as for group 2, 48 h cytokines stimulation
Group 5	HCE-T only, as for group 3, TGF-β1 10 ng/ml 1 h treatment, followed 48 h cytokines stimulation
Group 6	HCE-T/MSC, untreated control
Group 7	HCE-T/MSC, 24 h TGF-β1 10 ng/ml treatment
Group 8	HCE-T/MSC, 48 h TGF-β1 10 ng/ml treatment

**Table 5:** Groups for TGF-β1 signaling pathway experiments.

Group 1	HCE-T only, untreated control
Group 2	HCE-T only, 24 h
	100 U/ml rh IFN-γ +100 U/ml rH TNF stimulation
Group 3	HCE-T/MSC1 h vehicle (DMSO) treatment followed 24 h
	[HCE-T 100 U/ml rH IFN-γ +100 U/ml rH TNF]
	[MSC 100 U/ml rR IFN-γ +100 U/ml TNF]
Group 4	HCE-T/MSC, treatment 1 h anti- TGF-β1 antibody (1 µg/ml) and SB-431542 (1 µM/ml), then 24 h [HCE-T 100 U/ml rH IFN-γ +100 U/ml rH TNF]
	[MSC 100 U/ml rR IFN-γ +100 U/ml rR TNF]
Group 5	HCE-T only, as for group 2, 48 h cytokines stimulation
Group 6	HCE-T/MSC, as for group 3, 1 h DMSO treatment, followed 48 h cytokines stimulation
Group 7	HCE-T/MSC, as for group 4, 1 h anti- TGF-β1 antibody (1 µg/ml) and SB-431542 (1 µM/ml) treatment, followed 48 h cytokines stimulation

### Immunology assays

#### 1. Flow cytometry

To characterize MSCs, cells were harvested by trypsinization, and were resuspended in either PE conjugated anti-CD29, FITC conjugated anti-CD44, anti-CD34, Alexa conjugated anti-CD45, or its isotype controls (Table 6). To detect the expression of CD54 (ICAM-1, MHC-I (HLA-ABC) and MHC-II (HLA-DR) on HCE-Ts, HCE-Ts were subjected to the cytokine treatment (Table 1) and then harvested by trypsinization. HCE-Ts were resuspended in either mouse anti-human Alexa 488 conjugated antibody CD54, MHC-I (HLA-ABC) and MHC-II (HLA-DR); or mouse anti-human HLA-G antibody (Table 6) for 1 h at 4°C. Cells were centrifuged through FBS, and resuspended in culture media for FCM analysis. To detect HLA-G expression, cells were further incubated with donkey anti-mouse Alexa Fluor 488 secondary antibody for 1 h at 4°C. Cells incubated with isotype hamster or mouse IgG were used as controls (Table 6). Flow cytometric analysis was then performed (Canto, Becton Dickinson, Sydney, Australia), and data was analyzed with Flow Jo software (Tree Star Inc, California, USA). Sample values were obtained by setting all isotype control values at channel 100. Graphed values were obtained by subtracting geometric means of isotype from experimental antibody labeled values. Statistics were undertaken as described below.

**Table 6:** Antibodies, conjugates and immunoreagents.

Primary antibodies and immunoglobulin (Ig) controls	Working dilutions concentrations	Applications	Manufacturer
PE anti-mouse/rat CD29	1∶50 1∶100	FCM IF	BioLegend, CA, USA
PE anti-rat CD44H	1∶50	FCM	BioLegend
FITC mouse anti-rat CD34	1∶5	FCM	Santa Cruz, CA, USA
Alexa Fluor 488 mouse anti-rat CD45	1∶50	FCM	BioLegend
Alexa Fluor 488 anti- human CD54 (ICAM-1)	1∶20	FCM	BioLegend
Alexa Fluor 488 anti-human HLA-A, B, C (MHC-I)	1∶20	FCM	BioLegend
Alexa Fluor 488 mouse anti-human HLA-DR (MHC-II)	1∶50	FCM	BioLegend
Purified anti-human HLA-G (MHC-Ib)	1∶50	FCM	BioLegend
Rabbit anti-NF-κB (p65)	1∶100	IF	Santa Cruz, CA, USA
	1∶5000	WB	
Mouse anti-IDO	1∶1000	WB	Millipore, MA, USA
Mouse anti-GAPDH	1∶10,000	WB	Hytest Ltd. Finland
Rabbit anti-histone H2B	1∶1000	WB	Cell Signaling, MA, USA
PE Armenian hamster IgG isotype control	1∶20	FCM	BioLegend
Alexa Fluor 488 mouse IgG1 isotype control (FC)	1∶20	FCM	BioLegend
FITC mouse IgG1 isotype control	1∶5	FCM	BioLegend
Mouse IgG	10 µg/ml	FCM	Jackson ImmunoResearch Laboratory
Rabbit IgG	4 µg/ml	IF	Jackson ImmunoResearch Laboratory, PA, USA
Secondary antibodies	Dilutions	Applications	Manufacturer
Donkey anti-mouse Alexa Fluor 488	1∶1000	FCM	Molecular Probes, CA, USA
Donkey anti-rabbit Alexa Fluor 488	1∶1000	IF	Molecular Probes, CA, USA
HRP conjugated goat anti-mouse IgG	1∶20,000	WB	Millipore
HRP conjugated goat anti-rabbit IgG	1∶25,000	WB	Millipore

FCM: flow cytometry; WB: Western blotting; IF: immunofluorescence staining.

#### 2. Immunofluorescent staining

This method was used to view NF-κB nuclear translocation. HCE-Ts were seeded on to glass cover slips placed in a 24 well plate (5×10^3^ cells/well). MSC were seeded on 6.5 µm diameter of Transwells (10^4^ cells/well). The groups and treatments were as described in Table 1. After the treatments, coverslips were fixed in 100% methanol (Ajax Finechem, Thermo Fisher Scientific Australia Pty Ltd) for 10 min at 4°C. Cells were blocked with 10% normal donkey serum (Zymed, Life Technologies, CA, USA)/PBS prior to incubation with rabbit anti-NF-κB or negative control rabbit IgG (Table 6) overnight at 4°C. After rinsing with PBS, cells were incubated with donkey anti-rabbit Alexa Fluor 488 (Table 6) and 1 µg/ml of Hoechst 33342 (Sigma-Aldrich) for 1 h at room temperature (RT). Immunolabelling was visualized and imaged using an inverted Olympus DP71 fluorescence microscope (Olympus, Center Valley, PA, USA).

#### 3. Enzyme-linked immunosorbent assay (ELISA)

Conditioned medium from each treatment group listed in Table 3 was collected and centrifuged at 400xg at 4°C for 10 min, and the supernatants were carefully collected and stored at −80°C prior to ELISA analysis. Measurement of the concentration of total TGF-β1 was performed with a TGF-β1 DuoSet ELISA kit (R&D Systems, Australia, Bio-Scientific Pty. Ltd.), while measurement of the concentration of active TGF-β1 was performed with a LEGEND MAX Free Active TGF-β1 ELISA Kit (BioLegend) according to the manufacturer's instructions. Briefly, standards and 100 µl of supernatants were loaded into the wells, and the absorbencies were measured at 450 nm using a Tecan Safire^2^microplate reader and analyzed by Magellan software (Tecan, Männedorf, Switzerland). All samples were tested in triplicate.

#### 4. Western blot

HCE-T cell cultures (Table 1, 4 and 5) were washed with cold PBS twice, and lysed in ice-cold RIPA buffer (Sigma-Aldrich) supplemented with complete mini-protease inhibitor cocktail tablet (Roche Diagnostics, Mannheim, Germany). Both cytoplasmic and nuclear extracts were prepared with a nuclear and cytoplasmic extraction kit (Millipore, Australia, Pty, Ltd) according to the manufacturer's instructions. The protein concentrations of the lysates were determined by QuantiPro BCA Assay Kit (Sigma-Aldrich) according to the manufacturer's instructions.

Cell lysates (15 µg) from each group were electrophoresed in 10% SDS-polyacrylamide gels, then transferred onto polyvinylidene difluoride (PVDF, 0.45 um) membranes (Invitrogen) as described previously [24]. Briefly, the membranes were blocked with 5% bovine serum albumin (BSA, Sigma), and then incubated with anti-NF-κB p65 (an isoform of NF-κB), anti-IDO, anti-histone H2B and anti-GAPDH antibodies (Table 6) at 4°C overnight. The blots were then incubated with horseradish peroxidase (HRP)–conjugated secondary antibodies at RT for 2 h, and developed using Enhanced chemiluminescence (ECL) Plus (Thermo Scientific) and visualized using a Syngene G Box system (Syngene, Cambridge, UK). Protein expression was determined using GeneTool software (version 4.03.02.0, Syngene, UK) and relative protein levels normalized to GAPDH or Histone 2B were used for comparisons.

### Statistical analysis

All experiments were repeated at least 3 times. Results were expressed as mean ± standard error of the mean (SEM). The statistical significance was assessed using GraphPad Prism 5 (GraphPad Software, La Jolla, CA, USA). One way analysis of variance (ANOVA) with Tukey's multiple comparison was used for multi-group comparisons, and a two-tailed unpaired Student's t test was used for comparisons between two groups, and a p value of <0.05 was considered statistically significant.

## Results

### Modulatory effects of MSC on IgSF molecule expression

**Figure 1:**
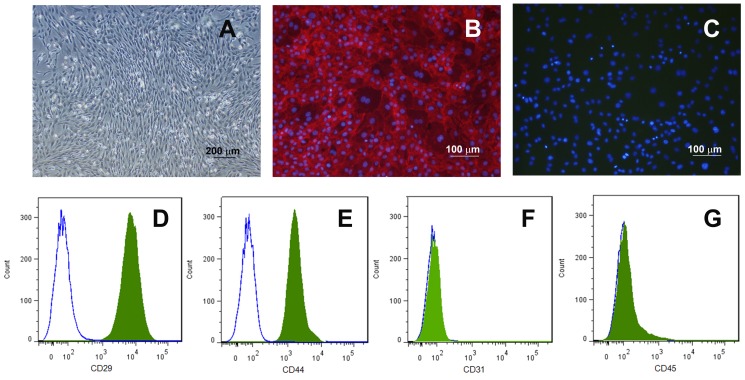
Immunophenotype of rat MSCs. Rat MSCs display spindle shape and reach confluent at 14 days post-isolation (**A**). More than 98% of the population are positive to the mesenchymal markers CD29 (**B, D**) and CD44 (**E**), and negative to the hematopoietic markers CD34 (**C, F**) and CD45 (**G**). Green shaded profile, antibodies staining; blue open profile, isotype control.

More than 98% of isolated primary rat MSC exhibiting the characteristic spindle shaped morphology were CD29^+^ and CD44^+^ with no CD34^+^ or CD45^+^ cells detected by flow cytometry (Figure 1). These cells have been demonstrated to be able to differentiated into corneal epithelial-like cells [25].

**Figure 2:**
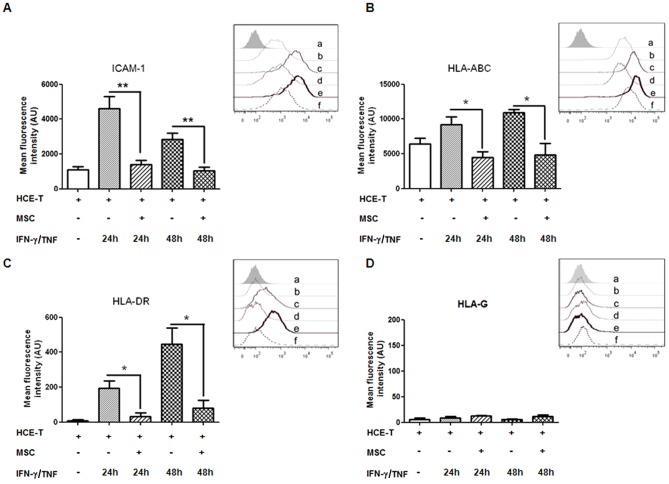
Modulatory effects of MSC on IgSF molecule expression. Bar graphs showing the relative expressions of ICAM-1 (**A**), HLA-ABC (**B**), HLA-DR (**C**) and HLA-G (**D**) on HCE-T cells, as determined by FCM. HCE-Ts were either untreated or treated for 24 h and 48 h with IFN-γ (100 U/ml) and TNF (100 U/ml) on monocultures and HCE-T/MSC co-cultures. The inserts are representative FCM histograms, and the abscissa represents log10 fluorescent intensity in arbitrary units (AU) [a. isotype control Alexa 488 conjugated mouse IgG; b. untreated HCE-T; c. 24 h IFN-γ/TNF treatment on HCE-T monoculture; d. 24 h IFN-γ/TNF treatment on HCE-T/MSC co-culture; e. 48 h combination cytokines stimulation on HCE-T monoculture; f. 48 h combination cytokine stimulation on HCE-T/MSC co-culture]. Data represents mean ± SEM of four separate experiments (n = 4). *: p<0.05, **: p<0.01. The “+” or “-“ symbol denotes the presence or absence of the denoted treatment or cell type at the left.

HCE-T constitutively expressed ICAM-1 and MHC-I, while HLA-DR and HLA-G was not expressed above isotype control levels (Figure 2 and inserts). HCE-T treated with IFN-γ (100 U/ml) and TNF (100 U/ml) showed a maximal 3-4-fold increase in ICAM-1 at 24 h, reducing to a 2-fold increase by 48 h, compared to untreated controls. MSC/HCE-T co-culture abrogated this up-regulation (p<0.01 Figure 2A), with ICAM-1 expression remaining at control levels at both 24 h and 48 h in these cultures. Similarly, co-culture abrogated the cytokine-induced increase in MHC-I at 24 h and 48 h (both p<0.05, Figure 2B) and the progressively increasing *de novo* HLA-DR expression at these time points (p<0.05, Figures 2C). In contrast, HLA-G expression on HCE-T was not modulated by cytokine stimulation or MSC co-culture (Figure 2D). The level of expression of these antigens in untreated HCE-T/MSC co-culture was similar to that of untreated HCE-T (data not shown in figure).

### Modulatory effect of MSC on cytokine-induced NF-κB activation

**Figure 3:**
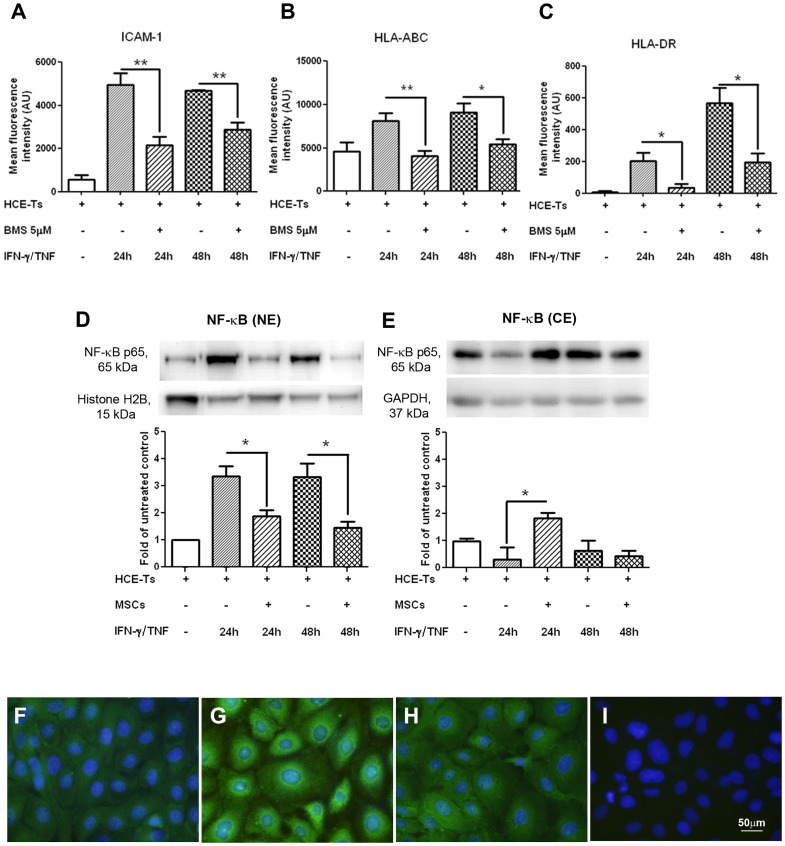
Blocked NF-κB activation inhibits cytokine-upregulated IgSF molecules expression, and MSC modulates cytokine-induced NF-κB nuclear translocation. A to C shows FCM analysis of HCE-T cells treated with BMS-345541 (5 µM), a NF-κB pathway inhibitor, significantly inhibited IFN-γ (100 U/ml) and TNF (100 U/ml) induced ICAM-1 (A), HLA-ABC (B), and HLA-DR (C) expression. Data represents mean ± SEM of four separate experiments (n = 4). *: p<0.05, **: p<0.01. D and E (upper panel) show representative bands of western blots for NF-κB p65 (65 kDa) in nuclear extracts (NE, D) and cytoplasmic extracts (CE, E). Histone H2B (15 kDa) and GAPDH (37 kDa) were used as the loading controls for nuclear and cytoplasmic extracts, respectively. Protein was prepared from HCE-T cells with or without IFN-γ/TNF stimulation, or HCE-T/MSC co-cultured with 24 h and 48 h IFN-γ/TNF treatment. The lower panel shows the fold changes of NF-κB within nuclear extracts and cytoplasmic extracts compared with the untreated group, using Histone H2B and GAPDH as loading controls. Data represents mean ± SEM of four separate experiments (n = 4). *: p<0.05. F to I show representative immunocytochemistry images. Untreated HCE-T cells displayed weak NF-κB nuclear and cytoplasmic staining (F); NF-κB expression showed marked nuclear and perinuclear translocation after 24 h IFN-γ/TNF stimulation of HCE-T monocultures (G); NF-κB perinuclear and nuclear translocation decreased at 24 h in IFN-γ/TNF in stimulated HCECs/MSC co-cultures (H); HCE-T monolayer showed no obvious staining with rabbit IgG (I). The “+” or “−“ symbol denotes the presence or absence of the denoted treatment or cell type at the left.

As many of the pro-inflammatory effects of IFN-γ and TNF are induced via NF-κB translocation, we investigated whether this pathway was modulated by MSC. As expected, the induced expression of ICAM-1 and MHC-I/MHC-II was significantly inhibited at 24 and 48 hour time points by HCE-T treatment with the NF-κB inhibitor, BMS-345541, confirming the dependence of up-regulation of all three molecules on NF-κB translocation (Figure 3A to C, p<0.05). Furthermore, Western blot of HCE-T nuclear extracts showed a 3-fold increase in nuclear translocation of NF-κB p65 protein at both 24 h and 48 h after cytokine treatment, compared to untreated controls (Figure 3D). However, co-culture with MSC significantly reduced translocation at both time points (both p<0.05, Figure 3D). There was no significant reduction of NF-κB p65 in cytoplasmic extracts (Figure 3E) after 24 h cytokine exposure compared to untreated controls. However, a significant increase in cytoplasmic retention was observed when co-cultured with MSC at this time point (p<0.05). Neither of the 48 h cytoplasmic values was significantly different from controls. Compared to control HCE-T (Figure 3F), IFN-γ/TNF treatment induced obvious perinuclear staining and nuclear translocation of NF-κB p65 by 24 h in HCE-T by immunohistochemistry (Figure 3G) and this was clearly reduced in HCE-T/MSC co-cultures (Figure 3H). Similar patterns of immunolabelling were observed at 48 h (not shown). HCE-T monolayers showed no obvious staining with rabbit IgG (Figure 3I).

### Effect of MSC on cytokine-induced IDO expression

**Figure 4:**
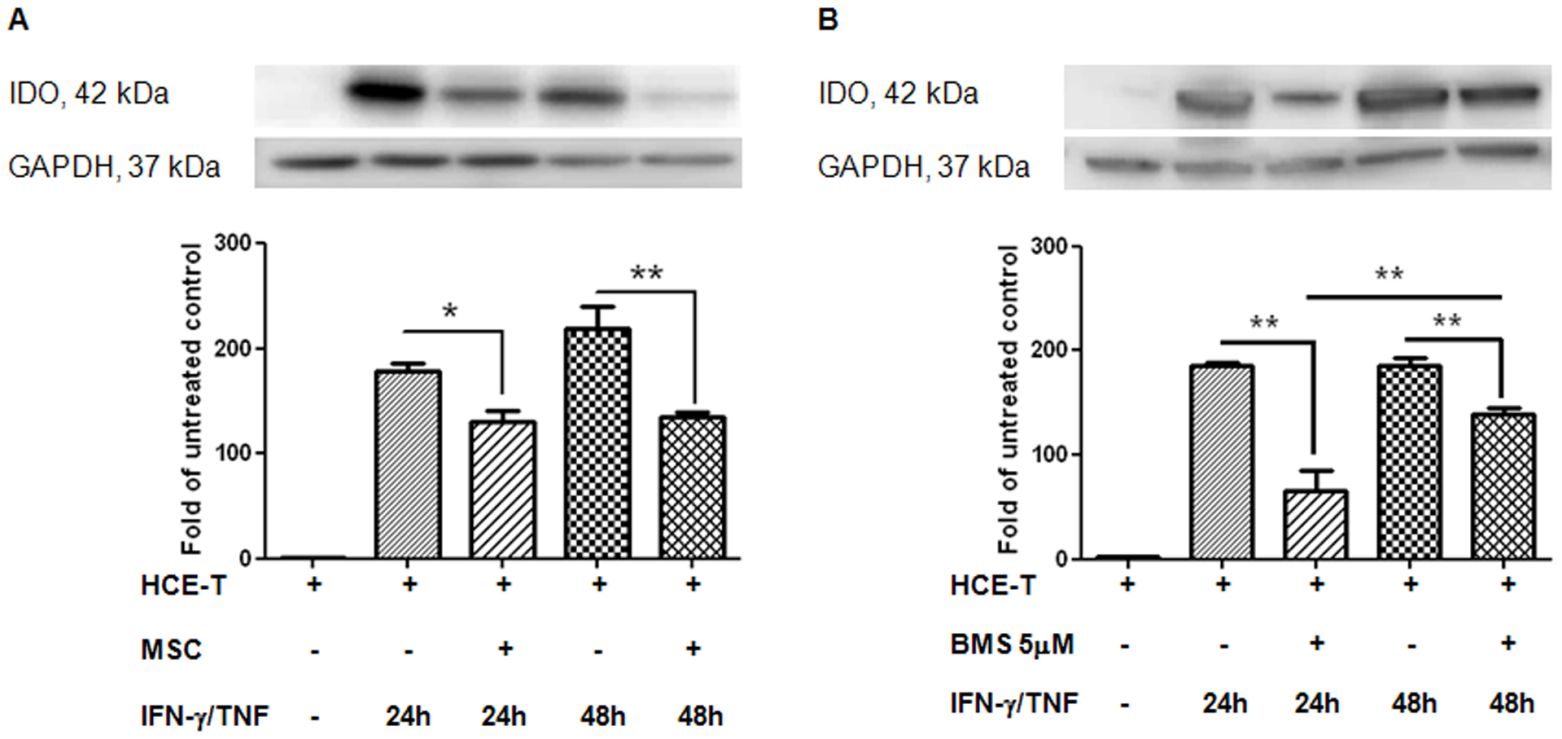
The modulatory effect of MSC on cytokine-induced IDO expression, and the association of NF-κB to the modulation. The upper panels are representative images of individual experiments. (**A**) Bar graphs show IDO expression in untreated HCE-T, HCE-T monocultures and HCE-T/MSC co-cultures stimulated for 24 h and 48 h with IFN-γ (100 U/ml) and TNF (100 U/ml), semi-quantified by densitometry with GAPDH as loading control. Combination treatment with cytokine remarkable induced IDO expression in HCE-T monocultures, while HCE-T/MSC co-cultured significantly attenuated this induction on 24 h (p<0.05) and 48 h (p<0.01). (**B**) Treatment with BMS-345541, a NF-κB pathway inhibitor, significantly reduced the cytokine-induced-IDO expression at both 24 h and 48 h (p<0.01). Data represents the results from three individual experiments (n = 3) ± SEM. *: p<0.05, **: p<0.01. The “+” or “−“ symbol denotes the presence or absence of the denoted treatment or cell type at the left.

We next investigated the effect of MSC on cytokine-induced IDO expression. Western blot analysis showed almost undetectable IDO expression in untreated HCE-T (Figure 4A). Treatment with IFN-γ/TNF progressively increased IDO expression by approximately 180- and 220-fold at 24 h and 48 h, respectively, however, this was significantly inhibited by MSC co-culture at 24 h (p<0.05) and 48 h (p<0.01) (Figure 4A). Furthermore, similar to the effect on cytokine-induced IgSF molecules, treatment with BMS-345541 significantly reduced IFN-γ/TNF-induced IDO expression at both 24 h and 48 h (p<0.01, Figure 4B). Interestingly, however, this treatment had a significantly greater effect at 24 h than 48 h (p<0.01, Figure 4B), suggesting IDO up-regulation may also be occurring via another pathway.

### The involvement of TGF-β1 in the modulatory effect of MSC on IDO induction

**Figure 5:**
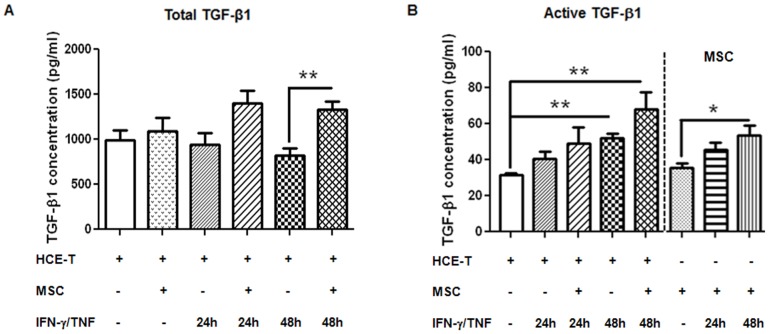
The effect of MSC on TGF-β1 secretion. The concentration of (**A**) total and (**B**) active TGF-β1 secretion in conditioned media collected from untreated HCE-T monocultures, MSC monocultures and HCE-T/MSC co-cultures, or HCE-T monocultures, MSC monocultures and HCE-T/MSC co-cultured with IFN-γ/TNF stimulation for 24 h and 48 h was measured by ELISA. (**A**) A similar level of TGF-β1 was detected from untreated HCE-T monocultures and HCE-T/MSC co-cultures. IFN-γ/TNF-stimulated HCE-T/MSC co-culture showed increased total TGF-β1 from HCE-Ts at 24 h (p = 0.052), and significantly increased at 48 h (P<0.01). (**B**) IFN-γ/TNF treatment significantly increased active TGF-β1 secretion from HCE-T (p<0.01) and MSC (p<0.05) monocultures at 48 h. Compared to untreated monocultures, IFN-γ/TNF-stimulated HCE-T/MSC co-culture showed a significant increase in active TGF-β1 after 48 h, (p<0.05). Data represents the results from three individual experiments (n = 3) ±SEM. *: p<0.05, **: p<0.01. The “+” or “−“ symbol denotes the presence or absence of the denoted treatment or cell type at the left.

Because of the immunosuppressive nature of TGF-β1 and since this is produced by MSC [26], [27], an ELISA was performed to evaluate the secretion of TGF-β1 in HCE-T/MSC co-cultures. There was no significant difference in total TGF-β1 production between untreated HCE-T monocultures and HCE-T/MSC co-cultures. Stimulation with IFN-γ/TNF over 48 h did not statistically increase the amount of total TGF-β1 in HCE-T monocultures. However, IFN-γ/TNF-stimulated HCE-T/MSC co-culture increased TGF-β1 from HCE-T by 24 h (p = 0.052), and significantly increased at 48 h (P<0.01, Figure 5A). Levels of active TGF-β1 were <5% of the total TGF-β1 and similar in untreated HCE-T and MSC monocultures (Figure 5B). IFN-γ/TNF treatment significantly increased active TGF-β1 secretion from HCE-T (p<0.01) and MSC (p<0.05) monocultures by 48 h. Compared to untreated monocultures, IFN-γ/TNF-stimulated HCE-T/MSC co-culture showed a significant increase in active TGF-β1 production after 48 h, [70.2±8.92 pg/ml (mean±SEM), p<0.01], levels were greater than IFN-γ/TNF-treated MSC or HCE-T monocultures (MSCs 53.8±5.1 pg/ml and HCE-T 51.8±2.9 pg/ml respectively) (Figure 5B).

**Figure 6:**
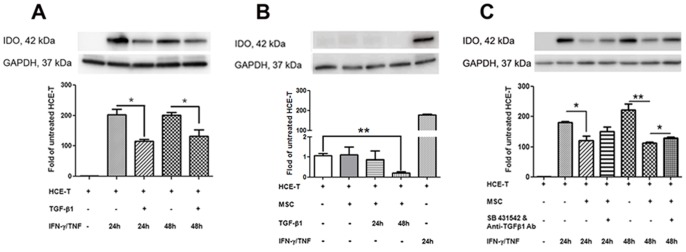
The involvement of TGF-β1 in the modulatory effect of MSCs on IDO induction. (**A**) Untreated HCE-T, HCE-T monocultures exposed to IFN-γ/TNF for 24 h and 48 h with or without 10 ng/ml TGF-β1 pre-treatment. The cytokines-induced IDO expression was significantly inhibited by TGF-β1 at 24 h and 48 h (p<0.05 at both time points). (**B**) HCE-T/MSC co-cultures without and with TGF-β1 10 ng/ml 24 h treatment express a similar low level of IDO as untreated HCE-T monocultures (less than 2 folds). The IDO expression is significant lower in the HCE-T/MSC co-cultures with 48 h TGF-β1 treatment compared to the untreated HCE-T monolayers (p<0.01). While IDO expression from HCE-Ts stimulated with IFN-γ/TNF for 24 h was more than 180 folds higher than untreated HCE-Ts. (**C**) Untreated HCE-T, HCE-T monocultures stimulated with IFN-γ/TNF for 24 h and 48 h, or HCE-T/MSC co-cultures stimulated by IFN-γ/TNF with or without combined SB-431542 (1 µM) and anti-TGF-β1 antibody (1 µg/ml) treatment. Blockade of the TGF-β1 signaling pathway in IFN-γ/TNF-treated MSC/HCE-T co-cultures using combined neutralizing anti-TGF-β1 antibody and SB-431542, a TGF-β receptor I blocker, significantly reversed the down-regulatory effect of MSC on IDO expression by 48 h (p<0.05). The upper panels are representative images of individual western blots for IDO (42 kDa) and GAPDH (37 kDa) expression in HCE-T cells. Each bar summarizes the data from 3 individual experiments (n = 3) ±SEM. *: p<0.05, **: p<0.01. The “+” or “−“ symbol denotes the presence or absence of the denoted treatment or cell type at the left.

To investigate the association between TGF-β1 and the modulatory effects of MSC on HCE-T, exogenous TGF-β1 was added to IFN-γ/TNF-treated HCE-T monocultures. Western blot analysis showed that IDO expression was significantly inhibited at 24 h and 48 h (p<0.05 at both time points, Figure 6A), while MSCs did not affect IDO expression on untreated HCE-Ts. Interestedly, IDO expression on HCE-T/MSC co-cultures treated with TGF-β1 for 48 h was significant lower than untreated HCE-T monocultures (p<0.01, Figure 6B). Furthermore, blockade of the TGF-β1 signaling pathway in IFN-γ/TNF-treated MSC/HCE-T co-cultures by a combination of neutralizing anti-TGF-β1 antibody and SB-431542, a TGF-β receptor I blocker, resulted in significant inhibition of the down-regulatory effect of MSC on IDO expression by 48 h (p<0.05, Figure 6C). However, this did not fully remove the effect of MSC co-culture, confirming that IDO is downregulated by more than one pathway in this system.

## Discussion

The effects of stem cells on corneal wound healing and epithelial regeneration have been widely discussed with respect to their potential for trans-differentiation [16]–[18]. However, focus has recently increased on the potential anti-inflammatory effects of MSC in these processes. Although the administration of MSC systemically or topically has a therapeutic effect in ocular surface wound healing by reducing local inflammation and neovascularization, mechanistic detail remains unclear [19]–[21], [28], [29]. To further elucidate this, we used an *in vitro* model to examine the anti-inflammatory and immunoregulatory effects of MSC on pro-inflammatory cytokine (IFN-γ/TNF)-stimulated human corneal epithelial cells.

The infiltration of inflammatory cells into the site of cell damage is an initial step in tissue injury that depends on the interactions between locally upregulated adhesion molecules such as ICAM-1 on tissue cells, and LFA-1 expressed on virtually immigrating leukocytes. Excessive inflammatory cell infiltration may lead to severe corneal damage, for example, via enzymes released from neutrophils [30], while neovascularization, mediated by VEGF from infiltrating neutrophils and macrophages, ultimately reduces function [31], [32]. In addition, epithelial MHC-I and MHC-II molecules could informally present antigen to T-lymphocytes that migrate to sites of corneal injury in adaptive responses due to infection or autoimmunity. Up-regulated MHC-I and MHC-II would increase the efficiency of this interaction and render epithelial cells more susceptible to T cell-mediated cytotoxicity [33]-[35]. Our results showed that MSC effectively abrogated IFN-γ/TNF-induced ICAM-1 and MHC-I and II expression on HCE-T. An inhibitory effect on ICAM-1 expression mediated by MSC has been observed in MSC/pulmonary endothelial cell co-cultures [36] and MSC/human umbilical endothelial cell (HUVECs) co-cultures [37]. To our knowledge, this is the first time inhibition of these molecules mediated by MSC has been observed on corneal epithelium.

In contrast to MHC-II, which was induced *de novo* by IFN-γ/TNF treatment in our study, HLA-G expression on HCE-T was neither induced *de novo* by these cytokines, nor affected by MSC co-culture. HLA-G is an important soluble factor for immunomodulation by MSC [38]. Immunosuppressive functions of HLA-G include inhibition of CD4^+^ and CD8^+^ T-cell proliferation, and impairment of natural killer cell activation and inhibition of CD4^+^ T-cell proliferation [38], [39]. Lack of responsiveness of HLA-G in our study may be related to a) the possible requirement for direct contact between MSC and HCE-T to modulate HLA-G expression on HCE-T and/or b) insufficient IFN-γ/TNF to induce HLA-G expression or c) refractoriness of the HCE-T cells in this study.

The transcription factor, NF-κB, regulates the expression of a large number of genes coding for cell surface molecules involved in inflammatory responses [34], [40]. Inhibition of NF-κB decreases the expression of molecules such as ICAM-1 and VCAM-1 on HUVEC and epithelial cells, and MHC-I and MHC-II on dendritic cells [41]–[43]. Suppression of NF-κB translocation and reduction of NF-κB mRNA have been demonstrated in MSC transplantation in myocardial infarction and in traumatic brain injury rat models [44], [45]. BMS-343341 prevents neutrophil infiltration in an animal model of spinal cord injury by inhibiting ICAM-1 expression and down-regulates TNF-induced ICAM-1 expression in HUVEC [46], [47]. NF-κB inhibition with BMS-343341 in our studies confirmed the NF-κB pathway dependence of IFN-γ/TNF-induced ICAM-1, MHC-I and MHC-II up-regulation, while MSC similarly, significantly reduced IFN-γ/TNF-induced NF-κB nuclear translocation in corneal epithelial cells. The mechanism by which MSC down-regulate NF-κB translocation in our model is unclear. Some studies have shown that TNF-α-stimulated gene/protein 6 (TSG-6) can inhibit nuclear translocation of NF-κB [45], [48], [49]. Increased TSG-6 with decreased NF-κB mRNA and protein has also been reported with MSC transplantation in a rat model of traumatic brain injury[45]. The role of TSG-6 in our model will be explored in future studies.

TGF-β1 is an important cytokine with potent immunoregulatory properties. It regulates the differentiation, proliferation and activation of lymphocytes, macrophages and dendritic cells and has immunosuppressive effects on T cells and neutrophils in a broad spectrum of pathological circumstances [50]. TGF-β1 is constitutively secreted by MSC, and the addition of anti-TGF-β1 antibody attenuated the suppressive effect of MSC on proliferation of peripheral blood lymphocytes [51]. Topical and systemic administration of MSC in animal models of chemical- or heat-induced corneal injury revealed increased levels of TGF-β1 mRNA and protein in the epithelial, stromal and endothelial layers of cornea [20], [28]. Our data show that IFN-γ/TNF stimulation over 48 h significantly increased active TGF-β1 secretion from MSC and HCE-T monocultures. In addition, the level of total TGF-β1 in 48 h cytokine-stimulated HCE-T/MSC co-cultures was increased compared to 48 h cytokine-stimulated HCE-T monocultures, indicating that the increase in total TGF-β1 was associated with the presence of MSC. TGF-β1 has been reported to suppress NF-κB activation [52], but we observed neither reduced NF-κB translocation in response to exogenous TGF-β1 nor down-regulation of the IgSF molecules tested (data not shown). On the other hand, TGF-β clearly down-regulated IDO expression in cytokine-stimulated HCE-T/MSC co-cultures.

IDO catalyses the breakdown of the essential amino acid, L-tryptophan, to kynurenine. As the initial and rate-limiting enzyme of the kynurenine pathway, the depletion of L-tryptophan locally inhibits T cell proliferation, while kynurenine is toxic to a range of immune cells [53]. IDO-dependent suppression of T-cell responses appears to function as a natural immunoregulatory mechanism. Our results show that IFN-γ/TNF stimulation significantly induced IDO expression on HCE-T and is consistent with published data that IFN-γ induces IDO expression and TNF acts synergistically [54]. Modulatory IDO production and activity is potentially a feature of MSC immunoregulation [55]. However, to our knowledge the effect of MSC on IDO expression induced in cells acted on by MSC has not been investigated. Contrary to our expectations, MSC significantly attenuated cytokine-induced IDO expression on HCE-T, while the inhibition of IDO by BMS-345541 confirmed the importance of the NF-κB pathway in its induction. Interestingly, the reduction of IDO expression was greater at 24 h than 48 h, suggesting that IDO up-regulation may be controlled by other pathways.

Exogenous TGF-β1 treatment of cytokine-stimulated HCE-T monoculture also significantly attenuated cytokine-induced IDO expression, while in cytokine-stimulated co-cultures, TGF-β receptor I blockade and neutralizing TGF-β1 antibody together substantially reversed the modulating effect of MSC on cytokine-induced IDO expression. Previous studies have shown that TGF-β inhibits IFN-γ-induced IDO activation in human monocyte-derived-macrophages [56], and abrogates IFN-γ-induced IDO expression in human skin and synovial fibroblasts *in vitro* at both the mRNA and protein levels [57]. Over expression of IDO decreases colonic epithelial cell proliferation and results in cell cycle arrest in lens epithelial cells, since tryptophan is crucial for epithelial cell replication [58], [59]. Our findings show that in MSC/HCE-T co-cultures, TGF-β1 down regulates IDO expression in corneal epithelial cells and that the modulatory effect of MSC involves both NF-κB and TGF-β1 signaling pathways in our model. The reduction of IDO expression by MSC may contribute to corneal re-epithelialization after injury.

Previous studies using MSC from heterogenic species to investigate corneal repair have used human MSC in animal models [19], [60]. In contrast, we used rat MSC with human corneal epithelium *in vitro*. Notwithstanding, our data corroborate these *in vivo* studies, further emphasizing the correspondence of these anti-inflammatory process between species.

There are limitations of this *in vitro* model. Clearly, the effect of MSC on local leukocyte recruitment into cytokine-stimulated tissue and the influence of IDO on corneal re-epithelization would be better investigated in an *in vivo* model. Nevertheless, studies to dissect out further potential mechanisms of MSC-mediated anti-inflammatory modulation of corneal repair and injury protection are warranted. Co-cultured HCE-T and MSC provide a useful *in vitro* model to study this.

In conclusion, our results suggest that MSC reduce the expression of adhesion molecules and immunoregulatory molecules on HCE-T stimulated with pro-inflammatory cytokines, via NF-κB signaling pathways. Increased TGF-β1 secretion in MSC and MSC/HCE-T co-cultures inhibited NF-κB activation and attenuated cytokine-induced IDO expression. These modulatory effects may provide an environment more favorable for corneal epithelial regeneration, further supporting the therapeutic potential of MSC in corneal injury recovery.

## Supporting Information

Figure S1 Hypothesis, experimental design and results of the study. Flowchart summarizes the hypothesis, experimental design and results of the current study.
(JPG)
