# Supplementary figures and images for: Immunomodulatory Effects of Bone Marrow-Derived Mesenchymal Stem Cells on Pro-Inflammatory Cytokine-Stimulated Human Corneal Epithelial Cells
Source: PLoS One. 2014 Jul 8;9(7):e101841. doi: 10.1371/journal.pone.0101841 (PMC4086952; doi:10.1371/journal.pone.0101841)

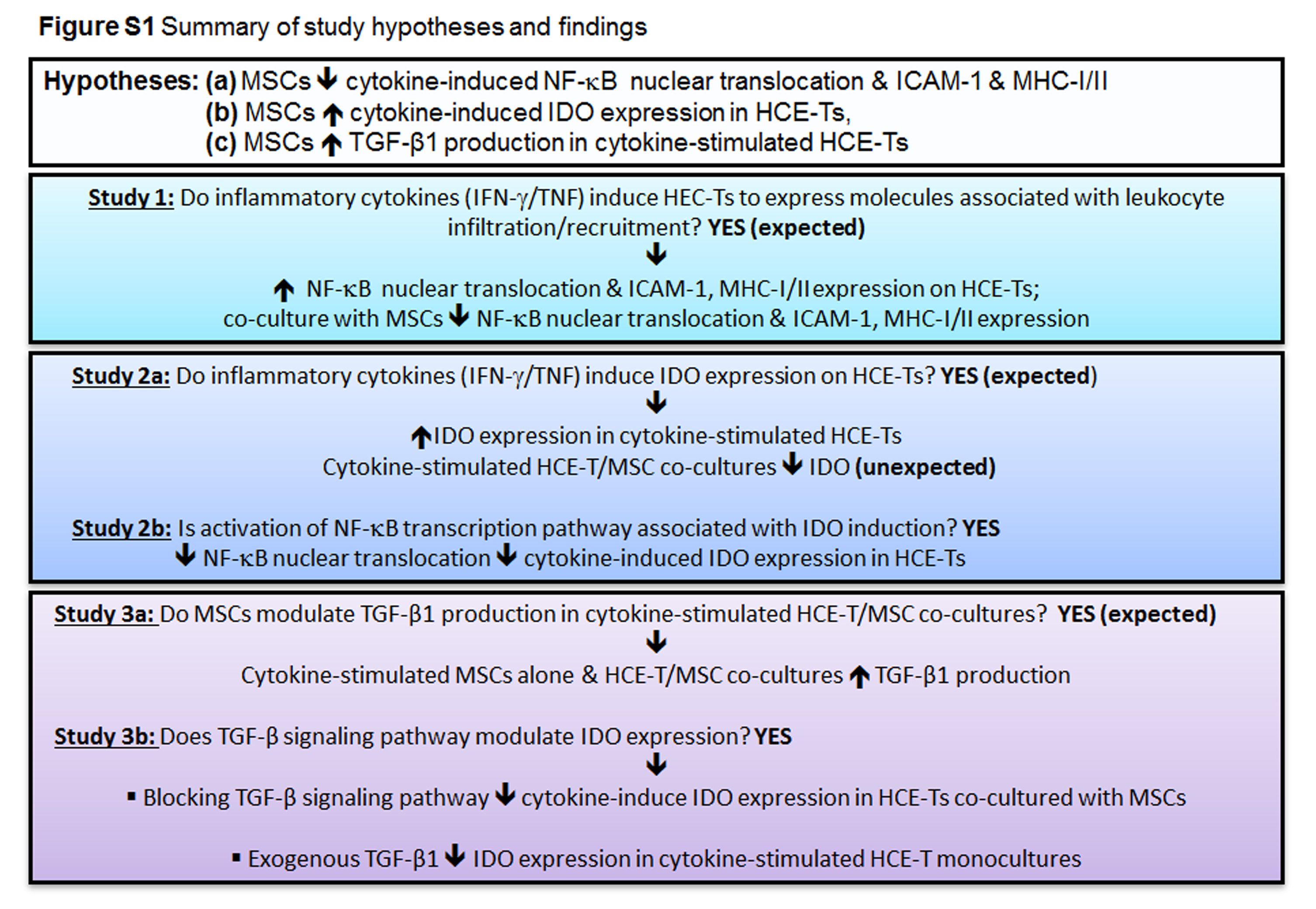

Supplement: Figure S1 — Hypothesis, experimental design and results of the study. Flowchart summarizes the hypothesis, experimental design and results of the current study. (JPG) [file pone.0101841.s001.jpg]
